# Contribution of proteomic studies towards understanding plant heavy metal stress response

**DOI:** 10.3389/fpls.2012.00310

**Published:** 2013-01-25

**Authors:** Zahed Hossain, Setsuko Komatsu

**Affiliations:** ^1^Department of Botany, West Bengal State UniversityKolkata, West Bengal, India; ^2^National Institute of Crop ScienceTsukuba, Japan

**Keywords:** antioxidant, heavy metal, HSPs, phytochelatins, proteomics, PR protein

## Abstract

Modulation of plant proteome composition is an inevitable process to cope with the environmental challenges including heavy metal (HM) stress. Soil and water contaminated with hazardous metals not only cause permanent and irreversible health problems, but also result substantial reduction in crop yields. In course of time, plants have evolved complex mechanisms to regulate the uptake, mobilization, and intracellular concentration of metal ions to alleviate the stress damages. Since, the functional translated portion of the genome plays an essential role in plant stress response, proteomic studies provide us a finer picture of protein networks and metabolic pathways primarily involved in cellular detoxification and tolerance mechanism. In the present review, an attempt is made to present the state of the art of recent development in proteomic techniques and significant contributions made so far for better understanding the complex mechanism of plant metal stress acclimation. Role of metal stress-related proteins involved in antioxidant defense system and primary metabolism is critically reviewed to get a bird’s-eye view on the different strategies of plants to detoxify HMs. In addition to the advantages and disadvantages of different proteomic methodologies, future applications of proteome study of subcellular organelles are also discussed to get the new insights into the plant cell response to HMs.

## INTRODUCTION

High-throughput OMICS techniques are extensively being exploited in recent times to dissect plants molecular strategies of heavy metals (HMs) stress tolerance. Plants growing in HMs contaminated environment have developed coordinated homeostatic mechanisms to regulate the uptake, mobilization, and intracellular concentration of toxic metal ions to alleviate stress damages. As the functional translated portion of the genome play a key role in plant stress response, proteomic studies provide us a finer picture of protein networks and metabolic pathways primarily involved in cellular detoxification and tolerance mechanism against HM toxicity.

By definition, elements having specific gravity above five are considered as HMs. Nevertheless, the term HM commonly refers to toxic metals, e.g., cadmium (Cd), copper (Cu), chromium (Cr), lead (Pb), zinc (Zn) as well as hazardous metalloids viz., arsenic (As), boron (B), which exert negative effects on plant growth and development (Hossain et al., 2012a).

Most of the HMs get their entry into plant root system via specific/generic ion carriers or channels (Bubb and Lester, 1991). The lack of specificity of transporters that are primarily involved in uptake of essential elements such as Zn^2+^, Fe^2+^, and Ca^2+^ allow the entry of Cd^2+^, Pb^2+^ (Welch and Norvell, 1999; Perfus-Barbeoch et al., 2002). Once HM ions enter the cell, cellular functions are affected by a wide range of actions. The negative impact of HM includes binding of HM ions to sulfhydryl groups of proteins, replacement of essential cations from specific binding sites, leading to enzyme inactivation and production of reactive oxygen species (ROS), resulting in oxidative damages to lipids, proteins and nucleic acids (Sharma and Dietz, 2009).

Over the last decade, extensive research on plants response to HM stress has been conducted to unravel the tolerance mechanism. Genomics technologies have been useful in addressing plant abiotic stress responses including HM toxicity (Bohnert et al., 2006). However, changes in gene expression at transcript level have not always been reflected at protein level (Gygi et al., 1999). An in-depth proteomic analysis is thus of great importance to identify target proteins that actively take part in HM detoxification mechanism.

Plant response to HM stress has been reviewed extensively over the past decade (Sanita Di Toppi and Gabbrielli, 1999; Cobbett, 2000; Ma et al., 2001; Cobbett and Goldsbrough, 2002; Hall, 2002; Maksymiec, 2007; Sharma and Dietz, 2009; Verbruggen et al., 2009; Yang and Chu, 2011; Hossain et al., 2012a). However, review articles on application of proteomics in analyzing cellular mechanism for HM tolerance are limited (Ahsan et al., 2009; Luque-Garcia et al., 2011; Villiers et al., 2011).

Current review represents the state of art of recent developments in proteomic techniques and significant contributions made so far to strengthen our knowledge about plants HM-stress response cascade at protein level. Special emphasis is given to highlight the role of metal stress-related proteins engage in HM ions sequestration, antioxidant defense system, and primary metabolism for deeper understanding of coordinated pathways involve in detoxification of HM ions within plant cells. Furthermore, future applications of proteome study of subcellular organelles are discussed to get the new insights into the plant cell response to HMs.

## QUANTITATIVE PROTEOMIC TECHNIQUES USED FOR ANALYSIS OF HM-RESPONSIVE PROTEINS

Conventional two-dimensional gel electrophoresis (2-DE) approach coupled with protein identification by mass spectrometry (MS) has been the most widely used proteomic technique for investigation of HM-induced alteration of plant proteome composition (**Table 1**). Protein extraction and purification from the HM-stressed tissue is the most crucial step in 2-DE approach, as the amount and quality of the extracted proteins ultimately determine the protein spot number, resolution, and intensity. Phenolic compounds, proteolytic and oxidative enzymes, terpenes, pigments, organic acids, inhibitory ions, and carbohydrates are some common interfering substances present in recalcitrant plant tissues. Inferior 2-D separation results due to proteolytic breakdown, streaking, and charge heterogeneity. Proteomic studies on plant response against HM stress have revealed that trichloroacetic acid/acetone precipitation (Patterson et al., 2007; Zhen et al., 2007; Kieffer et al., 2008; Alves et al., 2011; Hossain et al., 2012b,c) and phenol-based (Bona et al., 2007; Alvarez et al., 2009; Vannini et al., 2009; Lee et al., 2010; Ritter et al., 2010; Rodríguez-Celma et al., 2010; Ahsan et al., 2012; Sharmin et al., 2012) protocols are the effective protein extraction methods for obtaining high quality proteome map. Nevertheless, phenol-based method is the most appropriate in extracting glycoproteins, and produce high-resolution proteome map for recalcitrant plant tissues (Saravanan and Rose, 2004; Komatsu and Ahsan, 2009).

**Table 1:** Summary of functional proteomic analyses in response to heavy metal stress (2007–2012).

Metal	Plant (tissue)	Protein extraction buffer + precipitation	Protein solubilization/lysis buffer	Proteomic methodologies	IP	Major findings	Reference
Cd	*G. max* L. cvs. Harosoy (H), Fukuyutaka (F), CDH-80 (C) (leaf, root)	10% TCA, 0.07% 2-ME in acetone	8 M urea, 2 M thiourea, 5% CHAPS, 2 mM TBP, ampholytes (pH 3–10)	IPG, 2-DE, nanoLC-MS/MS, MALDI-TOF MS	32 (HL), 26 (FL), 44 (CL), 16 (R)	Activation of SOD, APX, and CAT ensures cellular protection from ROS mediated damages under cadmium stress; enhanced expression of molecular chaperones help in stabilizing protein structure and function, thus maintain cellular homeostasis.	Hossain et al. (2012b)
	*G. max* L. cv. Enrei (leaf)	10% TCA, 0.07% 2-ME in acetone	8 M urea, 2 M thiourea, 5% CHAPS, 2 mM TBP, ampholytes (pH 3–10)	IPG, 2-DE, nanoLC-MS/MS, MALDI-TOF MS	78	High abundance of Hsp70 helps BABA-primed plants to maintain normal protein functions; higher abundance of Prx indicates BABA potentiated antioxidant defense system to combat Cd stress.	Hossain et al. (2012c)
	*G. max* L. cv. Enrei, Harosoy (root microsome)	0.5 M Tris–HCl (pH 8.0), 2 mM EDTA, 2 mM DTT, 0.25 M sucrose, 1 mM PMSF + Tris–HCl saturated phenol	8.5 M urea, 2.5 M thiourea, 5% CHAPS, 1% DTT, 1% Triton X-100, 0.5% Biolyte (pH 5–8)	IPG, 2-DE, nanoLC-MS/MS	22	Up-regulation of proteins associated with Cd-chelating pathways and increased lignification of xylem vessels lead to low root-shoot translocation of Cd in cv. Enrei.	Ahsan et al. (2012)
	*L. esculentum* Mill cv. Tres Cantos (root)	phenol-saturated Tris–HCl 0.1 M (pH 8.0), 5 mM ME	8 M urea, 2% (w/v) CHAPS, 50 mM DTT, 2 mM PMSF, 0.2% (v/v) 3–10 ampholytes	IPG, 2-DE, MALDI-TOF-MS, LIFT TOF–TOF	27 (low Cd), 33 (high Cd)	Low Cd treatment (10 µM) activates glycolysis, TCA cycle and respiration; at high Cd (100 µM) major decreases in growth, a shutdown of the carbohydrate metabolism and decreases in respiration takes place.	Rodríguez-Celma et al. (2010)
	*O. sativa* L. cv.Dongjin (Root, leaf)**	0.5 M Tris–HCl (pH 8.0), 50 mM EDTA, 900 mM sucrose, 100 mM KCl, 2% ME, 1 mM PMSF + Tris-buffered phenol (pH 8.0)	7 M urea, 2 M thiourea, 4% CHAPS, 1 mM PMSF, 50 mM DTT, 0.5% IPG buffer	IPG, 2-DE, MALDI-TOF MS	18 (R) 19 (L)	ROS scavengers (GST, APX, NADH-ubiquinone oxidoreductase) primarily up-regulated in roots under Cd treatment, indicates prompt antioxidative response against oxidative stress damages.	Lee et al. (2010)
	*H. vulgare* L. var. Baraka (leaf mesophyll tonoplast)	Tonoplast proteins dissolved in iTRAQ dissolution buffer	–	iTRAQ labeling, MALDI-TOF/TOF MS	56	Candidate proteins like CAX1a and MRP-like ABC transporter play significant role in vacular Cd^2+^ transport, hence Cd^2+^ detoxification.	Schneider et al. (2009)
	*B. juncea *L. (Acc: PI 173874) (root)	Tris-buffered phenol (pH 8.8) and 600 mL of 0.1 M Tris–HCl with 10 mM EDTA, 0.4% v/v 2-ME, 0.9 M sucrose	DIGE solubilization buffer (7 M urea, 2 M thiourea, 4% w/v CHAPS, 0.2% w/v SDS, 10 mM Tris, pH 8.5), and 0.5 M bicine pH 8.4 with 0.09% w/v SDS (for iTRAQ Label)	IPG, 2-D DIGE, iTRAQ, nanoLC-MS/MS	102 (DIGE), 585 (iTRAQ)	*O*-acetylserine sulfhydrylase, glutathione-*S*-transferase and glutathione-conjugate membrane transporter play essential role in the Cd hyperaccumulation and tolerance of *B. juncea*.	Alvarez et al. (2009)
	*P. tremula *L. (leaf)	20% TCA and 0.1% (w/v) DTT in ice-cold acetone	Labeling buffer	IPG, 2-D DIGE, MALDI-TOF-TOF MS	125	Up-regulation of mitochondrial respiration provides energy and reducing power to cope with met al stress, photosynthesis comparatively less affected.	Kieffer et al. (2008)
Cu	*E. siliculosus* strains Es32, Es524 (algal tissue)**	1.5% w/v PVP, 0.7 M sucrose, 0.1 M KCl, 0.5 M Tris–HCl (pH 7.5), 250 mM EDTA, protease inhibitor, 2% v/v ME, 0.5% w/v CHAPS + phenol saturated Tris–HCl (pH 7.5)	7 M Urea, 2 M Thiourea, 4% w/v CHAPS, 60 mM DTT, 20 mM Tris–HCl (pH 8.8), Biolytes (pH 3–10)	IPG, 2-DE, MALDI-TOF MS	10 (Es32) 14 (Es524)	Copper stress leads to up-regulation of photosynthesis (PSII Mn-stabilizing protein of OEC33), glycolysis, and pentose phosphate metabolism; higher accumulation of HSP70 and vBPO for proper protein folding and ROS detoxification respectively.	Ritter et al. (2010)
	*O. sativa* L. Wuyunjing (germinating embryos)	50 mM Tris–HCl (pH 8.0), 1 mM EDTA, 1 mM dithiothreitol (DTT), and 1 mM PMSF + ice-cold acetone with 1 mM DTT	8 M urea, 4% CHAPS, 65 mM DTT, 0.2% (w/v) Biolytes (pH 3–10)	IPG, 2-DE, MALDI-TOF MS	16	First proteomic evidence that met allothionein and CYP90D2 (a putative small cytochrome P450) are Cu-responsive proteins in plants.	Zhang et al. (2009)
	*C. sativa *Var. Felina 34 (root)	0.5 M Tris–HCl (pH 7.5), 0.7 M sucrose, 50 mM EDTA, 0.1 M KCl, 10 mM thiourea, 2 mM PMSF/DMSO, 2% v/v ME + phenol saturated Tris–HCl (pH 8.8)	9 M urea, 4% w/v CHAPS, 0.5% Triton X-100, 20 mM DTT, 2% v/v IPG Buffer	IPG, 2-DE, LC-MS/MS	20	Copper induced aldo/keto reductase acts as copper chaperone reduce copper ions to Cu (I), promote PCs-mediated vacuolar transport; Suppression/no change in ROS scavenging enzymes.	Bona et al. (2007)
	*O. sativa* L. cv. Hwayeong (Germinating seeds)	0.5 M Tris–HCl (pH 8.3), 2% v/v NP-40, 20 mM MgCl_2_, 2% v/v ME, 1 mM PMSF, 1% w/v PVP + acetone	9.5 M urea, 2% v/v NP-40, and 2.5% v/v pharmalytes (pH 3–10: pH 5–8: pH 4–6.5 = 1:3.5:2.5)	IEF gel (tube gel), 2-DE, MALDI-TOF MS	25	Excess Cu induces oxidative stress thus hampering metabolic processes; up-regulation of antioxidant and stress-related regulatory proteins (glyoxalase I, peroxiredoxin) help to maintain cellular homeostasis.	Ahsan et al. (2007b)
B	*L. albus* cv. Rio Maior (root)**	0.06 M DTT, 10% (w/v) TCA in cold acetone with 0.06 M DTT	2 M thiourea, 7 M urea, 4% (w/v) CHAPS, 0.4% (v/v) TritonX-100, 0.06 MDTT, and 1%(v/v) IPG buffer 3–10 NL	IPG, 2-DE, LC-MS/MS	128	Proteins associated with energy (glycolysis, TCA cycle, oxidation–reduction), cell division, protein metabolic processes suppressed under B deficiency.	Alves et al. (2011)
	*H. vulgare* cvs. GP, Cp, Sh, Cp x Sh DH (Root, leaf)	50 mM phosphate buffer (pH 7.5), 20 mM KCl, 0.5 M Suc, 10 mM DTT, 0.2 mM PMSF, 10 mM EDTA, 10 mM EGTA + 10% (w/v) TCA in acetone	0.5 M TEAB (pH 8.5) containing 0.1% SDS	iTRAQ peptide tagging, MS/MS	139	Higher abundance of Iron deficiency sensitive2 [IDS2], IDS3, and methylthio-ribose kinase observed in B-tolerant barley is linked to siderophore production	Patterson et al. (2007)
As	*Anabaena* sp. PCC7120 (algal cells)	10 mM Tris–HCl (pH 8.0), 1.5 mM MgCl_2_, 10 mM KCl + 10% (w/v) TCA in acetone	7 M urea, 2 M thiourea, 4% CHAPS, 40 mM DTT, and 1.0% IPG buffer (4–7)	IPG, 2-DE, MALDI-TOF, and LC-MS	45	Up-regulations of PGK, FBA II, FBPase, TK, ATP synthase, Prx, Trx, oxidoreductase help to maintain normal glycolysis, PPP, and turnover rate of Calvin cycle, protect cells from oxidative stress, thereby helping As-stress acclimation.	Pandey et al. (2012)
	*O. sativa *L. cv. Dongjin (leaf)**	0.5 M Tris–HCl (pH 8.3), 2% (v/v) NP-40, 20 mM MgCl_2_, 2% (v/v) ME, 1 mM PMSF, 0.7 M sucrose + acetone precipitation	8 M urea, 1% CHAPS, 0.5% (v/v) IPG buffer pH 4–7, 20 mM DTT	IPG, 2-DE, MALDI-TOF MS, ESI-MS/MS	12	Energy and metabolism related proteins over expressed indicating higher energy demand under As stress; down-regulation of RuBisCO and chloroplast 29 kDa ribonucleoproteins lead to decreased photosynthesis.	Ahsan et al. (2010)
As (V and III)	*A. tenuis *(leaf)**	Glacial acetone containing 0.07% (v/v) 2-ME, 0.34% (w/v) plant protease inhibitor, and 4% (w/v) PVP	4% (w/v) CHAPS, 7 M urea, 2 M thiourea, 2% (w/v) DTT, 1% (w/v) pharmalytes pH 3–10, 1% (w/v) resolytes pH 6–9.5	IPG, 2-DE, MALDI-TOF MS	31	As treatment resulted in partial disruption of the photosynthetic processes with prominent fragmentation of the RubisCO.	Duquesnoy et al. (2009)
	*O. sativa *L. cv. Dongjin (root)	0.5 M of Tris–HCl (pH 8.3), 2% v/v NP-40, 20 mM MgCl_2_, 2% v/v ME, 1 mM PMSF, 0.7 M sucrose + acetone precipitation	8 M urea, 1% CHAPS, 0.5% v/v IPG buffer pH 4–7, 20 mM DTT	IPG, 2-DE, MALDI-TOF MS	23	Energy, primary metabolic pathways suppressed under stress; higher GSH content coupled with enhanced expressions of GR, SAMS, GSTs, CS, GR mitigate As-induced oxidative stress.	Ahsan et al. (2008)
Mn	*V. unguiculata *[L.] Walp. cvs TVu 91, TVu 1987 (leaf)**	700 mM sucrose, 500 mM Tris, 50 mM EDTA, 100 mM KCl, and 2% v/v ME + water saturated phenol	8 M urea, 2% w/v CHAPS, 0.5% v/v IPG buffer pH 3–11, 50 mM DTT	IPG, 2-DE, Nano-LC-MS/MS, ESI MS/MS	8	Lower abundance of chloroplastic proteins involved in CO_2_ fixation and photosynthesis indicate channelizing metabolic energy to combat the Mn-stress; coordinated interplay of apoplastic and symplastic reactions essential for stress response.	Führs et al. (2008)
Cr	*M. sinensis* cv. Kosung (root)**	0.5 M Tris–HCl, pH 8.3, 2% (v/v) NP-40, 20 mM MgCl_2_, 1 mM PMSF, 2% (v/v) ME, and 1% (w/v) PVP	8 M urea, 1% CHAPS, 0.5% (v/v) IPG buffer pH 4–7, 20 mM DTT	IPG, 2-DE, MALDI-TOF MS, MALDI-TOF/TOF MS	36	Novel accumulation of chromium-responsive proteins (e.g. IMPase, nitrate reductase, adenine phosphoribosyl transferase, formate dehydrogenase, putative dihydrolipoamide dehydrogenase) observed; Cr toxicity is linked to heavy met al tolerance and senescence pathways.	Sharmin et al. (2012)
	*P. subcapitata* strain Hindák (algal cells)	500 mM Tris–HCl (pH 8), 700 mM sucrose, 10 mM EDTA, 4 mM ascorbate, 0.4% ME, 0.2% Triton X-100 10%, 1 mM PMSF, 1 µM Leupeptin, 0.1 mg/mL Pefabloc + water saturated phenol	7 M urea, 2 M thiourea, 4% CHAPS, 50 mg/mL DTT	IPG, 2-DE, LC-ESI-MS/MS	16	Cr-stress target photosynthetic proteins (RuBisCO, RuBisCO activase, Light Harvesting Chla/b protein complex, stress related Chl a/b binding protein) identified; Cr also induces modulation of proteins involved in amino acids metabolism.	Vannini et al. (2009)
Al	*G. max* (L.) Merr cvs. BaXi 10, BenDi 2 (root)	10% (w/v) TCA in acetone containing 0.07% (w/v) DTT, 1% PVP	7 M urea, 2 M thiourea, 2% (w/v) CHAPS, 1% (w/v) DTT, and 2% Pharmalyte pH 3–10	IPG, 2-DE, MALDI-TOF MS	30	Chaperones, PR 10, phytochrome B, GTP-binding protein, ABC transporter ATP-binding protein either newly induced or up-regulated, facilitate stress/defense, signal transduction, transport, protein folding, gene regulation, primary metabolisms.	Zhen et al. (2007)
	*O. sativa *L. cv. Xiangnuo 1 (XN1) (root)	40 mM Tris-base, 5 M urea, 2 M thiourea, 2% w/v CHAPS, 5% w/v PVP, and 50 mM DTT + ice-cold acetone with 0.07% (w/v) DTT	5 M urea, 2 M thiourea, 4% w/v CHAPS, 2% v/v IPG buffer, 40 mM DTT	IPG, 2-DE, MALDI-TOF/TOF MS, MALDI-TOF-MS	17	Antioxidation and detoxification lead by up regulation of Al-responsive proteins (Cu–Zn SOD, GST, SAMS 2), ultimately related to sulfur metabolism. CS, a novel Al-induced protein, play key role in Al resistance.	Yang et al. (2007)

*BABA, β -aminobutyric acid; CS, cysteine synthase; CHAPS, 3-[(3-cholamidopropyl) dimethylammonio]-1-propanesulfonate; Cp, Clipper; FBA II, fructose bisphosphate aldolase II; FBPase, fructose 1,6 bisphosphatase; GP, Golden Promise; IP, number of identified proteins; PPP, pentose phosphate pathway; Prx, peroxiredoxin; PGK, phosphoglycerate kinase; SAMS, *S*-adenosylmethionine synthetase; Sh, Sahara; TK, transketolase; Trx, thioredoxin; TBP, tributylphosphine; TEAB, triethylammonium bicarbonate; TSPP, tyrosine-specific protein phosphatase proteins; vBPO, vanadium-dependent bromoperoxidase.*

As compared to classical staining procedure of 2-DE gel using CBB or silver staining, advanced fluorescence two-dimensional difference gel electrophoresis (2-D DIGE) proteomic approach is now being used which allows comparison of the differentially expressed proteins of control and HM-stressed tissue on one single gel (Kieffer et al., 2008; Alvarez et al., 2009). DIGE is basically a gel-based method where proteins were labeled with fluorescent dyes (CyDyes – Cy2, Cy3, and Cy5) prior to electrophoresis. With the advancement of technology multiplexed isobaric tagging (iTRAQ) of peptides has allowed comparative, quantitative analysis of multiple samples. This second generation gel free proteomic approach has been well exploited for gaining comprehensive understanding of plants response to Cd and B (Patterson et al., 2007; Alvarez et al., 2009; Schneider et al., 2009).

## PLANT STRATEGIES OF HM TOLERANCE

**FIGURE 1:**
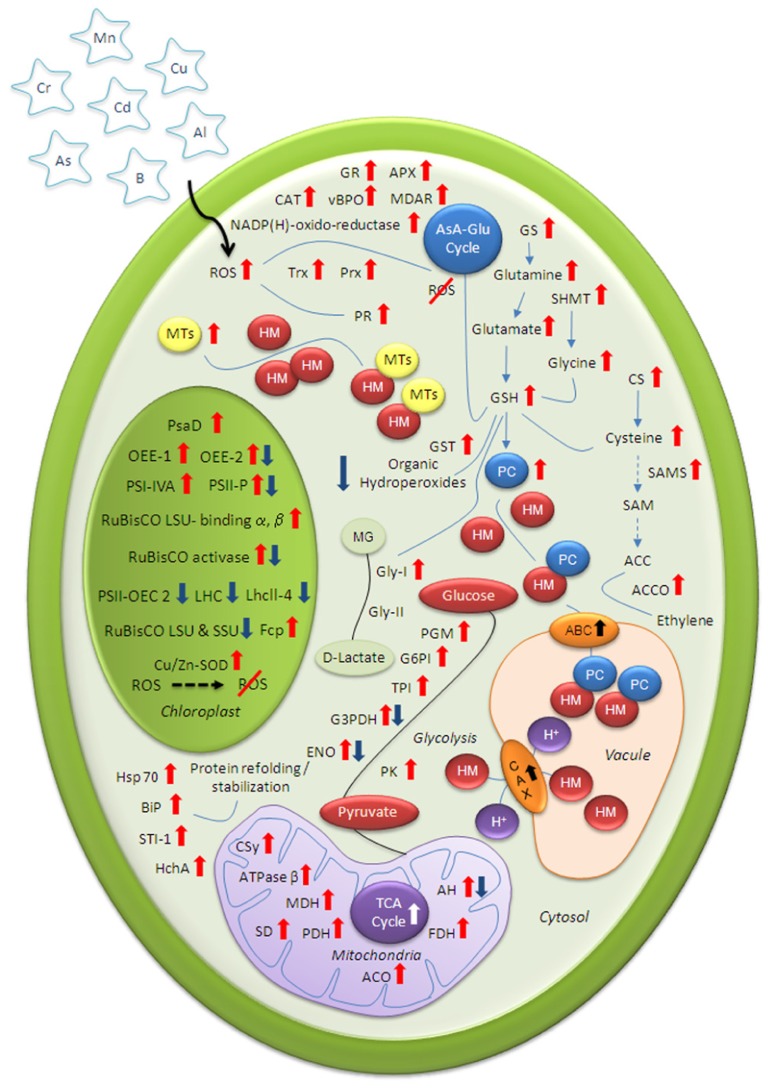
**Schematic illustration of various cellular mechanisms for mitigating heavy metal (HM) stress**. Information about highlighted proteins gathered from published proteomic articles related to plant HM-toxicity. Up and down arrows indicate HM-induced increase and decrease protein abundance respectively. ATPase β, ATP synthase subunit beta; AH, aconitate hydratase; AsA-Glu, ascorbate glutathione; APX, ascorbate peroxidase; ACC, 1-aminocyclopropane-1-carboxylic acid; ACO, aconitase; CAT, catalase; CAX, cation/proton exchanger; CS, cysteine synthase; CSy, citrate synthase; ENO, enolase; FDH, formate dehydrogenase; G3PDH, glyceraldehyde-3-phosphate dehydrogenase; GR, glutathione reductase; Gly-I, glyoxalase I; GS, glutamine synthetase; GSH, reduced glutathione; LHC, light harvesting complex; LhcII-4, light-harvesting chlorophyll-a/b binding protein; LSU, large subunit; MTs, metallothioneins; MG, methylglyoxal; MDAR, monodehydroascorbate reductase; MDH, malate dehydrogenase; OEE, oxygen-evolving enhancer protein; PC, Phytochelatin; Prx, peroxidoxin; PR, pathogenesis-related; PDH, pyruvate dehydrogenase; PSII-OEC 2, photosystem II oxygen-evolving complex protein 2; PS, photosystem; ROS, reactive oxygen species; RuBisCO, ribulose-1,5-bisphosphate carboxylase oxygenase; SD, succinate dehydrogenase; SAM, S-adenosylmethionine; SSU, small subunit; Trx, thioredoxin; TPI, triosephosphate isomerase; TCA, tricarboxylic acid.

In course of time, higher plants have evolved sophisticated mechanisms to regulate the uptake, mobilization, and intracellular concentration of HM ions (**Figure 1**). Apart from the plasma membrane exclusion method, the most common way to protect the cell from the adverse effects of HMs includes synthesis of membrane transporters and thiol-containing chelating compounds for vacuolar sequestration. Furthermore, increased abundance of defense proteins for effective ROS scavenging and molecular chaperones for re-establishing normal protein conformation help HM-stressed plants to maintain redox homeostasis. Modulations of vital metabolic pathways – photosynthesis and mitochondrial respiration – further help the stressed plant to produce more reducing power to compensate high-energy demand of HM challenged cells.

### COMPLEXATION, CHELATION, AND COMPARTMENTATION OF HMs WITHIN CELL

One of the important plant strategies of detoxifying HMs within cell is to synthesize low molecular weight chelators to minimize the binding of metal ions to functionally important proteins (Verbruggen et al., 2009). The thiol-containing chelating compounds strongly interact with HM, thus reducing free HM ions from cytosol and hence limiting HM toxicity (Cobbett and Goldsbrough, 2002). The phytochelatins (PCs) and metallothioneins (MTs), the two best characterized cysteine-rich HM binding protein molecules, play crucial roles in HM tolerance mechanism (Cobbett and Goldsbrough, 2002).

Phytochelatins synthesized from glutathione (GSH) by the enzyme PC synthase readily form complexes with HM in the cytosol and to facilitate their transport into vacuoles (Grill et al., 1989; **Figure 1**). Although PCs synthesis found to be induced in presence of most of the studied HMs, modulation of proteins, amino acids involved in PC biosynthesis have been the most widely studied in response to Cd. Our recent comparative proteome analysis of high and low Cd accumulating soybeans has revealed enhanced expression of glutamine synthetase (GS) under Cd stress. The enzyme GS is involved in the synthesis of GSH through glutamate biosynthesis pathway (Sarry et al., 2006; Semane et al., 2010). The enhanced expression of GS leads to more GSH formation (Hossain et al., 2012b). Induction of GSH synthesis implies higher metal binding capacity as well as enhanced cellular defense mechanism against oxidative stress (Verbruggen et al., 2009). Since GSH is the precursor of PC, enhanced expression of GS helps the cell to synthesize and accumulate more PC to detoxify cytosolic Cd^2+^. Our finding is in agreement with previous reports of up-regulation of GS in response to Cd (Kieffer et al., 2008; Hradilova et al., 2010; Semane et al., 2010; Ahsan et al., 2012). In contrast, sharp decline in GS abundance has been reported in Cd-stressed rice roots (Lee et al., 2010).

Ahsan et al. (2012) exploited proteomic technique in combination of metabolomics for deeper understanding of PC-mediated detoxification of Cd^2+^ in soybean roots. Comparative analysis revealed that proteins (GS beta 1) and amino acids (glycine, serine, glutamic acid) associated with Cd chelating pathways are highly active in low root-to-shoot Cd translocating cultivar. In addition, proteins involved in lignin biosynthesis were shown to be increased under stress. Proteomic findings suggest that translocation of Cd ions from the root to the aerial parts might be prevented by the increased xylem lignifications.

The PC biosynthetic pathway has been finely dissected in Cd-exposed *Arabidopsis thaliana* cells using protein and metabolite profiling (Sarry et al., 2006). At high Cd concentration global pool of GSH decreased dramatically with the increase in dipeptide γGlu-Cys, suggesting high cellular demand of GSH for sustaining PC [(γGlu-Cys)_n_-Gly] synthesis.

Alvarez et al. (2009) implemented two quantitative proteomics approaches – 2-D DIGE and iTRAQ – to find out the relation between Cd^2+^ sequestration and thiol metabolism. Both techniques identified an increased abundance of proteins involved in sulfur metabolism. Sulfite reductase and *O*-acetylserine sulfhydrylase, involved in reduction of sulfate to cysteine, were found to be overexpressed in Cd-treated *Brassica juncea* roots. Authors suggested that under Cd-stress, sulfate availability for synthesis of PCs and GSH may limit Cd tolerance. Significant inductions of GSH and PCs (PC_3_) in Cd-stressed rice roots further confirm the role of thiol-peptides in HM tolerance mechanism (Aina et al., 2007). Another proteomic study by Pandey et al. (2012) revealed higher abundance of cysteine synthase (CS) with higher contents of PCs and higher transcript of PC synthase in arsenic stressed *Anabaena* indicating their positive roles in As sequestration. Arsenic induced increases in GSH and PCs were also recorded in fronds of arsenic hyperaccumulator *Pteris vittata* (Bona et al., 2011). Interestingly, no such increase was evident in roots under As treatment. Proteomic results indicate that PCs could play role in As detoxification in *P. vittata* fronds only, but overall PC mediated detoxification is not the primary mechanism of As-tolerance in As hyperaccumulator, but to other adaptive mechanism. Up-regulation of proteins (CS and GSTs) and GSH pool involved in As detoxification has also been documented in proteomic study of As-stressed rice roots (Ahsan et al., 2008). Apart from Cd and As stress, CS and GSH also play essential role in Al adaptation for rice (Yang et al., 2007) and soybean (Zhen et al., 2007).

Unlike PCs, proteomics-based report on HM-induced alterations of MTs is very limited. Zhang et al. (2009) for the first time identified MT-like proteins from Cu-stressed germinating rice seed embryo. A number of gene expression studies have shown that MT genes are involved in Cu homeostasis and tolerance in *Arabidopsis* (Murphy and Taiz, 1995) and *Silene vulgaris* (van Hoof et al., 2001). Plant MTs not only play vital role in chelating Cu through the Cys thiol groups but are also considered as a potent scavenger of ROS (Cobbett and Goldsbrough, 2002; Wang et al., 2004).

The final step of HM detoxification involves sequestering of either free HMs or PCs-HMs complexes into cell vacuoles (Hall, 2002). This accumulation is mediated by tonoplast-bound cation/proton exchanger, P-type ATPase and ATP-dependent ABC transporter (Salt and Rauser, 1995; Hall, 2002). Transporters are also situated in plasma membrane and facilitate transport of HMs into apoplast. As the vacuoles or apoplasts have limited metabolic activity, accumulations of HMs in these compartments reduce the toxic effects of HMs (Schneider et al., 2009). The iTRAQ analysis of Cd-exposed barley leaf mesophyll tonoplast proteome led to the identification of ~50 vacuolar transporters, that include vacuolar ATPase subunits, MRP-like ABC transporter and two novel CAX transporters (CAX1a and CAX5) and one Al-activated malate transporter protein (Schneider et al., 2009). Induction of these transporters especially cation/proton exchanger 1a and ABC transporter assure Cd^2+^ transport into vacuole (Aina et al., 2007). Further proteomic study by Lee et al. (2010) revealed induction of vacuolar proton-ATPase in rice roots and leaves indicating their positive role in Cd detoxification through vacuolarisation.

### HM-INDUCED OXIDATIVE STRESS AND ALTERATION OF REDOX HOMEOSTASIS

Cellular ROS generation gets accelerated upon exposure to HM stress. HMs (Cu, Fe, Cr) that are directly involved in cellular redox reaction lead to ROS generation known as redox active, while redox inactive HMs (Cd, Al, As, Ni) trigger oxidative stress by depleting cells major thiol-containing antioxidants and enzymes, disrupting electron transport chain or by inducing lipid peroxidation (Ercal et al., 2001; Hossain et al., 2012a). The excess intracellular ROS level alters protein structure by inducing oxidation of both protein backbone and amino acid side chain residues (Villiers et al., 2011). To counter stress, plants have evolved robust antioxidant defense mechanism comprised of both enzymatic and non-enzymatic components (Hossain et al., 2012d).

Most of the proteomic research done so far on HM-related toxicity revealed positive correlation between tolerance and increased abundance of scavenger proteins. Within plant cells, SOD constitutes the first line of defense against ROS. It plays pivotal role in cellular defense against oxidative stress, as its activity directly modulates the amount of ${\text{O}}_{2}^{•-}$ and H_2_O_2_, the two important Haber–Weiss reaction substrates. The excess ${\text{O}}_{2}^{•-}$ generated under HM-stress usually disproportionate into H_2_O_2_ by the action of SOD, which is then metabolized by the components of the ascorbate–GSH cycle. Higher expressions of SOD isoforms (Cu/Zn-SOD, Fe-SOD) have been documented in plants exposed to excess Cd (Kieffer et al., 2008, 2009; Alvarez et al., 2009; Farinati et al., 2009; Semane et al., 2010; Hossain et al., 2012b) and Al (Yang et al., 2007). Interestingly, root proteome analysis of Cd-exposed *B. juncea* revealed up-regulation of Fe-SOD while down regulation of Cu/Zn SOD (Alvarez et al., 2009). Ascorbate peroxidase (APX), peroxidase (POD), and catalase (CAT) are involved in scavenging H_2_O_2_, hence protecting cell membrane from hydroxyl radical-induced lipid peroxidation (Barber and Thomas, 1978). The scavenging roles of APX, POD, and CAT have been documented in several proteomic studies related to Cd stress (Sarry et al., 2006; Aina et al., 2007; Kieffer et al., 2008; Lee et al., 2010; Hossain et al., 2012b) and As (Pandey et al., 2012) toxicity. Interestingly, excessive Cu (Bona et al., 2007), Cr (Sharmin et al., 2012) treatments or B deficiency (Alves et al., 2011) lead to decreased abundance of APX and POD. The detected suppression of POD is in accordance with the decrease in POD reported in maize roots treated with Al (Wang et al., 2011).

The abundance of another antioxidant enzyme of ascorbate–GSH cycle, monodehydroascorbate reductase (MDAR) was found to be increased in response to Cd (Sarry et al., 2006; Alvarez et al., 2009). MDAR helps to scavenge monodehydroascorbate radical and by doing this it generates dehydroascorbate (DHA), the oxidized form of ascorbate. Up-regulation of MDAR assures production of DHA, the substrate of dehydroascorbate reductase (DHAR) enzyme that catalyzes reduction of DHA to AsA (reduced ascorbate). In contrary, shoot proteome analysis of *Arabidopsis halleri* has shown decreased expression of MDAR in response to Cd, Zn, and rhizosphere microorganisms (Farinati et al., 2009). This down-regulation is also evident in roots of *Lupinus albus* undergoing long-term B deficiency (Alves et al., 2011). Decreased MDAR abundance in HM-stressed plants might indicate non-enzymatic disproportionation of monodehydroascorbate into AsA, essential for maintenance of balanced redox status (Hossain et al., 2009). Yet another well documented antioxidant found to be up-regulated under HM stress is peroxiredoxin (Prx). The Prx is basically a thiol peroxidase with multiple functions. It (a) detoxifies hydroperoxides; (b) plays essential role in enzyme activation and redox sensoring; (c) acts as molecular chaperone similar to HSPs; (d) induces cell signaling (; Jang et al., 2004; Barranco-Medina et al., 2009). Prx was found to be induced under Cd (Sarry et al., 2006; Ahsan et al., 2007a; Hossain et al., 2012b) and As (Requejo and Tena, 2006; Pandey et al., 2012) stress.

Plants are also equipped with some additional defense proteins, shown to be up-regulated by HM stress. This group includes thioredoxin (Trx), Trx-dependent peroxidase, NADP(H)-oxido-reductase and glyoxylase I (Gly I). Trx is known to suppress apoptosis as well as supplies reducing equivalents to antioxidants (Hishiya et al., 2008). Excess Cu treatment seems to down-regulate the abundance of Trx and Trx-POD in germinating rice embryo (Zhang et al., 2009) and *Cannabis sativa* roots (Bona et al., 2007) respectively. However, enhanced expression of Trx was found to be helpful in mitigating oxidative stress in As-treated *Anabaena* (Pandey et al., 2012).

Methylglyoxal (MG), a cytotoxic by-product of glycolysis generally accumulated in cell in response to environmental stresses including HM (Espartero et al., 1995). MG readily interacts with nucleic acids and proteins causing alteration of function (Yadav et al., 2005). Detoxification of MG through glyoxalase pathway involves active participation of GSH and Gly I and Gly II enzymes. Up-regulation of Gly I was found to help the germinating rice seedlings in detoxifying MG under Cd (Ahsan et al., 2007a) and Cu (Ahsan et al., 2007b). Higher Gly I abundance was also reported in Cd + Zn + microorganisms treated *A. halleri* shoots (Farinati et al., 2009). Proteomic study also highlighted enhanced expression of NADP(H)-oxido-reductase by Cd (Sarry et al., 2006; Lee et al., 2010) and As (Pandey et al., 2012). This protein is a vital component of plants second line of defense, protecting cells from HM-induced oxidative damages.

Plants tolerance against HMs is often attributed to steady state of GSH pool for its multifunctional activities in PC synthesis, MG detoxification, ROS scavenging through ascorbate–GSH cycle, GSTs mediated decomposition of toxic compounds as well as stress signaling (**Figure 1**). Within GSH cycle, glutathione reductase (GR) acts as a rate limiting enzyme that catalyzes reduction of oxidized glutathione (GSSG) to GSH (reduced glutathione) and with the help of DHAR it maintains high AsA/DHA ratio necessary for tight control of HM-induced ROS scavenging. The delicate balance between GSH and GSSG is critical for keeping a favorable redox status for the detoxification of H_2_O_2_. Higher abundance of GSTs has been observed in response to Cd (Alvarez et al., 2009; Lee et al., 2010), As (Ahsan et al., 2008; Pandey et al., 2012), Cu (Zhang et al., 2009). Findings of Ahsan et al. (2008) revealed increased activity of GST-omega in rice roots following exposure of AsV, indicating the probable role of GST-omega in inorganic arsenic biotransformation and metabolism. The authors also suggested that depletion in GSH content may be associated with high rate of PCs synthesis thus detoxification of As through compartmentalization or due to down-regulation of enzymes of GSH biosynthetic pathways such as GR and CS. The HM-induced PCs synthesis coupled with GSH depletion is in agreement with earlier studies by Sarry et al. (2006) and Di Baccio et al. (2005).

Proteomic analyses strongly indicate that accumulation of defense proteins chiefly enzymatic components of ascorbate–GSH cycle, POD, CAT, GSH, GSTs, Gly I, Prx, Trx help cells to mitigate HM-induced oxidative stress by scavenging ROS.

### MOLECULAR CHAPERONES

Protein dysfunction is an inevitable consequence of a wide range of adverse environmental conditions including HM toxicity. Molecular chaperones/heat-shock proteins (HSPs) are responsible for protein stabilization, proper folding, assembly, and translocation under both optimum and adverse growth conditions (Wang et al., 2004). In our study, enhanced abundance (>2-fold) of HSP70 protein was detected in leaves of high Cd-accumulating soybean cultivar Harosoy while low Cd-accumulating cv. Fukuyutaka exhibited decreased expression (Hossain et al., 2012b). Cd-induced up-regulation of HSP70 is also evident in response to various HMs including Cd (Kieffer et al., 2009; Hradilova et al., 2010; Rodríguez-Celma et al., 2010), Cr (Sharmin et al., 2012), and B deficiency (Alves et al., 2011). Ahsan et al. (2007a) reported increase of DnaK-type molecular chaperone BiP and chaperone protein HchA in germinating rice seedlings exposed to acute Cd toxicity. Al-stress also is known to induce one LMW-HSP and three DnaJ-like proteins in Al-stressed soybean (Zhen et al., 2007). To sum up, HSPs/chaperones play pivotal role in combating HM stress by re-establishing normal protein conformation and hence, cellular homeostasis.

### HM-INDUCED ALTERATION OF PROTEINS INVOLVED IN PHOTOSYNTHESIS AND ENERGY METABOLISM

Down-regulation of photosynthetic machinery is a known phenomenon of Cd stress. Low abundance of proteins involved in photosynthetic electron transport chain and Calvin cycle has been reported in Cd-exposed *Populus* (Kieffer et al., 2008, 2009; Durand et al., 2010) and *Thlaspi* (Tuomainen et al., 2006). Pioneer proteomic work by Hajduch et al. (2001) of rice leaves exposed to HMs revealed drastic reduction in abundance/fragmentation of large and small subunits of RuBisCO (LSU and SSU), suggesting complete disruption of photosynthetic machinery by HM stress. This decrease in RuBisCO has also been documented in other HMs toxicity like As (Duquesnoy et al., 2009) and Cd (Kieffer et al., 2008). Proteomic analysis for other toxic HMs like As-exposed leaf proteome of *Agrostis tenuis* has shown total disruption of RuBisCO LSU and SSU along with oxygen-evolving enhancer protein 1 and oxygen evolving protein 2 in response to 134 μM As(V) treatment (Duquesnoy et al., 2009). Potassium dichromate treatment had similar effects on algal RuBisCO LSU and some antenna proteins namely light harvesting Chl a/b protein complex. However, Vannini et al. (2009) reported higher abundance of RuBisCO activase in *Pseudokirchneriella subcapitata* under chromate treatment. Interestingly, in our proteomic experiment with Cd-exposed soybean, increased abundance of RuBisCO LSU-binding protein subunits alpha and beta, RuBisCO activase, oxygen-evolving enhancer protein 1 and 2, NAD(P)H-dependent oxido-reductase, photosystem I and II-related proteins were evident (Hossain et al., 2012b). Enhanced expressions of proteins involved in photosystem I, II, and Calvin cycle might be an adaptive feature to overcome the Cd injury in soybean. This increased abundance is in accordance with the findings of Semane et al. (2010), who also reported increase of photosynthetic protein abundance in leaves of *Arabidopsis* treated with mild Cd stress. In our opinion, contribution of high photosynthetic assimilates into respiration would help plants to yield more energy needed to combat the Cd^2+^ stress.

To maintain the normal growth and development under stressed environment, plants need to up regulate metabolic pathways such as glycolysis and tricarboxylic acid (TCA) cycle. Detailed analysis of HM toxicity-related proteomic works has shown higher abundance of glycolytic enzymes phosphoglycerate mutase (PGM), glucose-6-phosphate isomerase (G6PI), triose phosphate isomerase (TPI), glyceraldehyde-3-phosphate dehydrogenase (G3PDH), enolase (ENO), and pyruvate kinase (PK) in response to Cd (Sarry et al., 2006; Kieffer et al., 2008; Rodríguez-Celma et al., 2010; Hossain et al., 2012b), Cr (Labra et al., 2006). However, down-regulation of G3PDH was reported in As-treated rice roots (Ahsan et al., 2008) and roots of *Lupinus albus* under B deficiency (Alves et al., 2011). Similarly, Cu-treated *Cannabis* roots exhibited down-regulation of another glycolytic enzyme ENO, the metalloenzyme that catalyzes penultimate step of glycolysis – conversion of 2-phosphoglycerate to phosphoenolpyruvate (Bona et al., 2007).

Like glycolysis, enzymes of TCA cycle citrate synthase (CS), succinate dehydrogenase (SD), malate dehydrogenase (MDH), aconitase (ACO), aconitate hydratase (AH) were found to be up-regulated under Cd stress (Sarry et al., 2006; Kieffer et al., 2009; Rodríguez-Celma et al., 2010; Semane et al., 2010; Hossain et al., 2012b; **Figure 1**). In contrast, suppressions of several AH isoforms were evident in long-term B deficiency (Alves et al., 2011). Overall, up-regulation of glycolysis and TCA cycle might help the stressed plant to produce more reducing power to compensate high-energy demand of HM challenged cell.

### ACCUMULATION OF PR PROTEINS IN RESPONSE TO HM STRESS

Plant cells trigger some common defense machineries whenever they encounter a biotic or abiotic stress. Accumulation of PR proteins is one of such plant defense strategies and often associated with systemic acquired resistance (SAR) against a wide range of pathogens (Van Loon, 1997; Durrant and Dong, 2004). Using the 2-DE approach, Elvira et al. (2008) successfully identified different PR protein isoforms (viz. PR-1, β-1,3-glucanases PR-2, chitinases PR-3, osmotin-like protein PR-5, peroxidases PR-9, germin-like protein PR-16, and NtPRp27-like protein PR-17) in *Capsicum chinense* leaves and additionally resolved their specific accumulation pattern in both the compatible and incompatible PMMoV–*C. chinense* interactions. Apart from the assigned role in plant defense against pathogenic constraints, PR proteins also play key role in adaptation to stressful environments including HM toxicity (Hensel et al., 1999; Rakwal et al., 1999; Van Loon and Van Strien, 1999; Hajduch et al., 2001; Akiyama et al., 2004; Edreva, 2005). Kieffer et al. (2008) documented marked increase in abundance of PR proteins class I chitinases (PR-3 family), several β-1,3-glucanases (PR-2 family), and thaumatin-like protein (PR-5 family) in Cd-exposed poplar leaves. Endo-1,3-beta-glucanase, a class 2 PR protein, also found to be induced in rice roots under short-term Cd stress (Lee et al., 2010). Higher abundance of PR proteins under HM as documented in many proteomic studies is in accordance with previous transcriptomic analysis of mercuric chloride-treated *Zea mays* leaves (Didierjean et al., 1996). Like Cd stress, PR-10 and LIR18B protein (both belong to PR-10 family), and an acidic chitinase (PR-8 family) were *de novo* expressed under B deficiency (Alves et al., 2011). Stress-induced increase in ROS level has been shown to induce PR protein accumulation (Jwa et al., 2006). Treatment with excess Cu increased abundance of two PR proteins (PR-10a and putative PR proteins) in germinating rice embryos (Zhang et al., 2009). Analysis of the *Vigna unguiculata* leaf apoplast proteome using 2-DE and LC-MS/MS also revealed accumulation of several PR-like proteins glucanase, chitinase, and thaumatin-like proteins in response to excess Mn supply (Fecht-Christoffers et al., 2003). Transgenic tobacco overexpressing pepper gene CABPR1 encoding basic PR-1 protein showed enhanced resistance against HMs as well as pathogen stresses (Sarowar et al., 2005). These transgenic lines exhibited significant decline in total POD activity, suggesting that overexpression of CABPR1 in tobacco cells altered redox balance. Although, the precise role of PR proteins in combating HM stress is not yet clearly understood, the authors suggested that the induced redox imbalance might lead to H_2_O_2_ accumulation, triggering stress tolerance cascade. Several *in vitro* experiments have demonstrated that PR proteins display additional functions related to growth and development by modulating signal molecules (Kasprzewska, 2003; Liu and Ekramoddoullah, 2006). However, further proteomic investigations need to be undertaken to resolve the underlying molecular mechanism of PR proteins mediated plants HM tolerance.

## CONCLUSION AND FUTURE PROSPECTS

The present review outlines the impact of HMs stresses on plant proteome constituents. Most of the investigations done so far primarily highlighted the differential expression of proteins involved in plant defense and detoxification pathways, namely ROS scavenging, chelation, compartmentalization. In addition, accumulation of PR proteins and modulation of plants vital metabolic pathways CO_2_ assimilation, mitochondrial respiration in maintaining steady state of reducing power and energy required for combating HM-induced stress has been discussed in detail. Careful analysis of published proteomic works on HM toxicity has revealed that classical 2-DE coupled with MS-based protein identification has been the most widely used proteomic technique in investigating plant HM tolerance at organ/whole plant level. These proteomic findings have enriched us for deeper understanding plants HM tolerance mechanism.

The cellular mechanism of sensing stress and transduction of stress signals into the cell organelle represent the initial reaction of plant cells toward any kind of stress including HM. Communication through intracellular compartments plays a significant role in stress signal transduction process that finally activates defense gene cascade (Hossain et al., 2012d). To dissect the underlying molecular mechanism of how a plant cell modulates its protein signature to cope with stress, in depth study on organelle proteome would be of great contribution toward development of HM-tolerant crops.

As the PCs mediated HM-ion detoxification pathway ends in sequestration of PC-HM complexes into vacuole through various transporter proteins present in tonoplast membrane, more research on vacuole proteome needs to be undertaken for identification and characterization of novel metal transporter proteins responsible for cytoplasmic efflux of transition metal cations into vacuole. Legendary work by Schneider et al. (2009) on quantitative detection of changes in barley leaf mesophyll tonoplast proteome using advanced gel free iTRAQ method has enriched our knowledge about contribution of vacuolar transporters to Cd^2+^ detoxification. Plasma membrane proteome should be another target of future proteomic research on HM stress, as it acts as a primary interface between the cellular cytoplasm and the extracellular environment, thus playing a vital role in stress signal perception and transduction. Furthermore, transporter proteins present in cell membrane have importance in up-taking HM-ions into the cell. As most of the organelle membrane proteins are hydrophobic in nature, MS-based gel free system would be the most promising technique for identification of such proteins.

Plants response to multiple HMs would be another interesting area of future proteomic research (Sharma and Dietz, 2009). This could shed some light on cross talk between different HM stress signal pathways.

Heavy metal-induced protein oxidation study through redox proteomic approach has been the focus of much interest. More initiatives in this topic need to be taken as PTM/redox modification of proteins provides fundamental information about HM toxicity mechanism and biomarker discovery (Dowling and Sheehan, 2006; Braconi et al., 2011).

In summary, we believe that more research on sub-proteome-based HM approach would provide new insights into plants HM-stress response mechanism. HM-induced novel marker proteins would further enable us to design HM-tolerant transgenic crops.

## Conflict of Interest Statement

The authors declare that the research was conducted in the absence of any commercial or financial relationships that could be construed as a potential conflict of interest.

## Abbreviations

CBB: coomassie brilliant blue
2-DE: two-dimensional polyacrylamide gel electrophoresis
GS: glutamine synthetase
GSH: glutathione
GST: glutathione *S*-transferase
IEF: isoelectric focusing
LC: liquid chromatography
MS: mass spectrometry
MTs: metallothioneins
PCs: phytochelatins
pI: isoelectric point
PR: pathogenesis related
ROS: reactive oxygen species
SOD: superoxide dismutase.
